# Publisher Correction: Early preclinical detection of prions in the skin of prion-infected animals

**DOI:** 10.1038/s41467-019-08648-6

**Published:** 2019-02-04

**Authors:** Zerui Wang, Matteo Manca, Aaron Foutz, Manuel V. Camacho, Gregory J. Raymond, Brent Race, Christina D. Orru, Jue Yuan, Pingping Shen, Baiya Li, Yue Lang, Johnny Dang, Alise Adornato, Katie Williams, Nicholas R. Maurer, Pierluigi Gambetti, Bin Xu, Witold Surewicz, Robert B. Petersen, Xiaoping Dong, Brian S. Appleby, Byron Caughey, Li Cui, Qingzhong Kong, Wen-Quan Zou

**Affiliations:** 1 Department of Pathology, Case Western Reserve University School of Medicine, Cleveland, 44106 OH USA; 2 Department of Neurology, The First Hospital of Jilin University, Changchun, 130021 Jilin Province The People’s Republic of China; 3 Laboratory of Persistent Viral Diseases, Rocky Mountain Laboratories, National Institute of Allergy and Infectious Diseases (NIAID), National Institutes of Health (NIH), Hamilton, 59840 MT USA; 4 National Prion Disease Pathology Surveillance Center, Case Western Reserve University School of Medicine, Cleveland, 44106 OH USA; 5 Department of Otolaryngology, The First Affiliated Hospital of Xi’an Jiaotong University, Xi’an, 710061 Shanxi Province The People’s Republic of China; 6 Department of Biochemistry, Virginia Polytechnic Institute and State University, Blacksburg, 24061 Virginia USA; 7 Department of Physiology and Biophysics, Case Western Reserve University School of Medicine, Cleveland, 44106 OH USA; 8 Foundational Sciences, Central Michigan University College of Medicine, Mount Pleasant, 48859 MI USA; 9 State Key Laboratory for Infectious Disease Prevention and Control, National Institute for Viral Disease Control and Prevention, Chinese Center for Disease Control and Prevention, Beijing, 102206 The People’s Republic of China; 10 Department of Neurology, University Hospitals Cleveland Medical Center, Case Western Reserve University School of Medicine, Cleveland, 44106 OH USA; 11 National Center for Regenerative Medicine, Case Western Reserve University School of Medicine, Cleveland, 44106 OH USA

Correction to: *Nature Communications;* 10.1038/s41467-018-08130-9; Published online Jan 16 2019.

The original version of this Article contained errors in the author affiliations.

Affiliation 2 incorrectly read ‘Department of Neurology, The First Hospital of Jilin University, Changchun 130021 Jilin Province, China.’

Affiliation 5 incorrectly read ‘Department of Otolaryngology, The First Affiliated Hospital of Xi’an Jiaotong University, Xi’an 710061 Shanxi Province, China’

Affiliation 9 incorrectly read ‘State Key Laboratory for Infectious Disease Prevention and Control, National Institute for Viral Disease Control and Prevention, Chinese Center for Disease Control and Prevention, Beijing 102206, China.’

This has now been corrected in both the PDF and HTML versions of the Article.

