# Supplementary material for: Early preclinical detection of prions in the skin of prion-infected animals
Source: Nat Commun. 2019 Jan 16;10:247. doi: 10.1038/s41467-018-08130-9 (PMC6335425; doi:10.1038/s41467-018-08130-9)
Supplement: Supplementary file 1 — Supplementary Information [file 41467_2018_8130_MOESM1_ESM.pdf]

Early preclinical detection of prions in the skin of prion-infected animals

Zerui Wang[1,2†], Matteo Manca[3†], Aaron Foutz[4†], Manuel V. Camacho[1†], Gregory J. Raymond[3], Brent Race[3], Christina D. Orru[3], Jue Yuan[1], Pingping Shen[1,2], Baiya Li[1,5], Yue Lang[1,2], Johnny Dang[1], Alise Adornato[1], Katie Williams[3], Nicholas R. Maurer[1], Pierluigi Gambetti[1], Bin Xu[6], Witold Surewicz[7], Robert B. Petersen[1,8], Xiaoping Dong[9], Brian S. Appleby[1,4,10], Byron Caughey[3]*, Li Cui[2]*, Qingzhong Kong[1,4,9,11]*, Wen-Quan Zou[1,2,4,9,10,11]*

[1]Department of Pathology, Case Western Reserve University School of Medicine, Cleveland, Ohio, USA.

[2]Department of Neurology, The First Hospital of Jilin University, Changchun, Jilin Province, the People's Republic of China.

[3]Laboratory of Persistent Viral Diseases, Rocky Mountain Laboratories, National Institute of Allergy and Infectious Diseases (NIAID), National Institutes of Health (NIH), Hamilton, MT, USA.

[4]National Prion Disease Pathology Surveillance Center, Case Western Reserve University School of Medicine, Cleveland, OH, USA.

[5]Department of Otolaryngology, The First Affiliated Hospital of Xi'an Jiaotong University, Shaanxi Province, The People's Republic of China.

[6]Department of Biochemistry, Virginia Polytechnic Institute and State University, Blacksburg, Virginia, USA

[7]Department of Physiology and Biophysics, Case Western Reserve University School of Medicine, Cleveland, OH, USA.

[8]Foundational Sciences, Central Michigan University College of Medicine, Mount Pleasant, MI, USA.

[9]State Key Laboratory for Infectious Disease Prevention and Control, National Institute for Viral Disease Control and Prevention, Chinese Center for Disease Control and Prevention, Beijing, The People's Republic of China.

[10]Department of Neurology, University Hospitals Cleveland Medical Center, Case Western Reserve University School of Medicine, Cleveland, OH, USA.

[11]National Center for Regenerative Medicine, Case Western Reserve University School of Medicine, Cleveland, OH, USA.

*To whom correspondence should be addressed: wxz6@case.edu, or qxk2@case.edu, or chuili1967@126.com, or bcaughey@nih.gov

[†]Contributed equally to this work.

Supplementary Figure 1

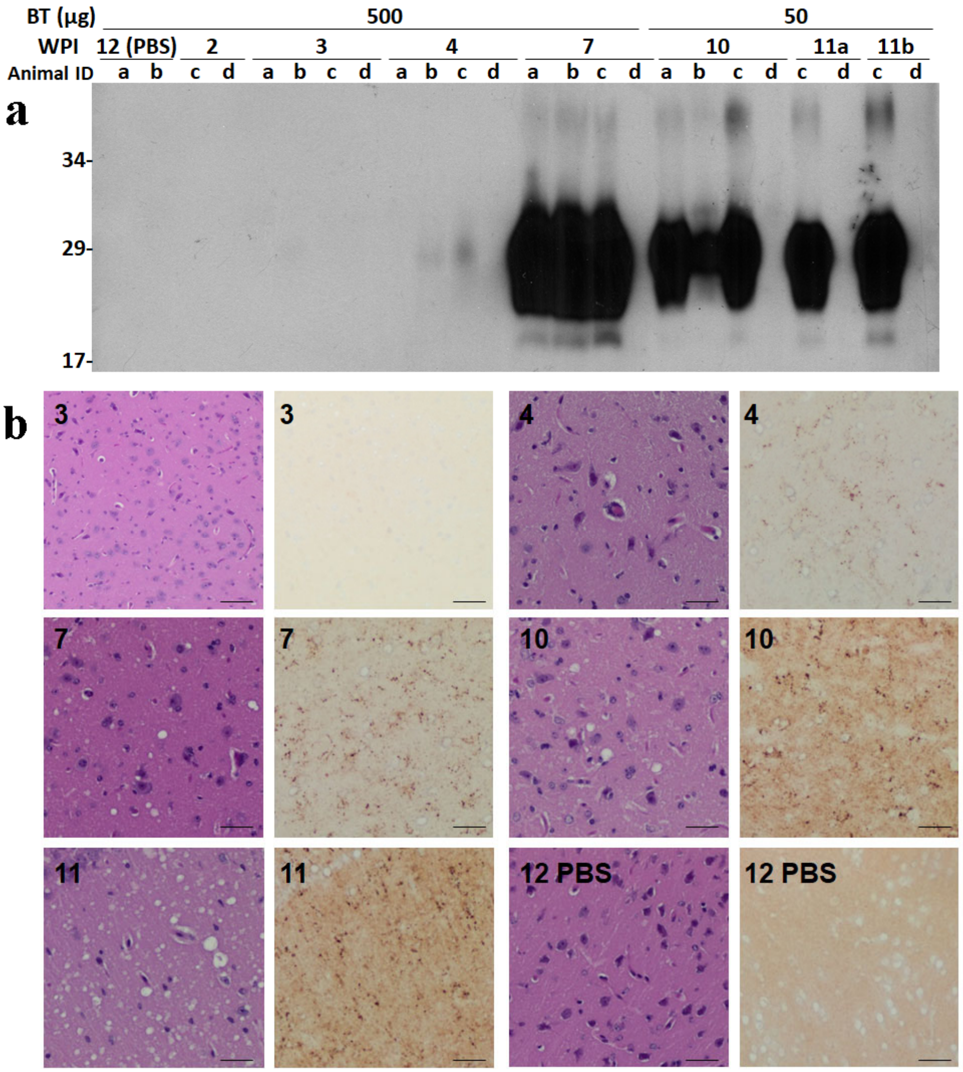

Supplementary Fig. 1 Detection of PrP^{Sc} and neuropathological changes in the brains of infected hamsters. (***a***) Representative Western blotting of PrP^{Sc} from the brain homogenates of infected hamsters sacrificed at different time points including 2, 3, 4, 7, 10, 11a, and 11b weeks post-inoculation (wpi) with brain homogenate containing 263K prion inoculum or PBS (12 wpi) as a negative control. Given expected low amounts of PrP^{Sc} in the brain of hamsters at the early stage of infection, 500 µg/lane of brain tissue (BT) was used for brain samples from hamsters

sacrificed at week 7 or earlier while 50 μg/lane from hamsters sacrificed at week 10 or later. Animal ID: a, b, and c represent hamsters that were inoculated with 263K prion while d represents hamsters that were inoculated with PBS at each time point. "11a" and "11b" represent animals with the same 11 wpi incubation time but housed in two different cages. All samples were treated with PK at 50 μg/ml. The blot was probed with 3F4. The molecular weight markers are shown in kDa on the left side of the blot. (***b***) Hematoxylin & Eosin (H&E) staining and immunohistochemistry of brain sections from hamsters sacrificed at different time points including 3, 4, 7, 10, and 11 wpi with 263K prions as well as 12 wpi with PBS negative controls. Bar = 50 μm.

Supplementary Figure 2

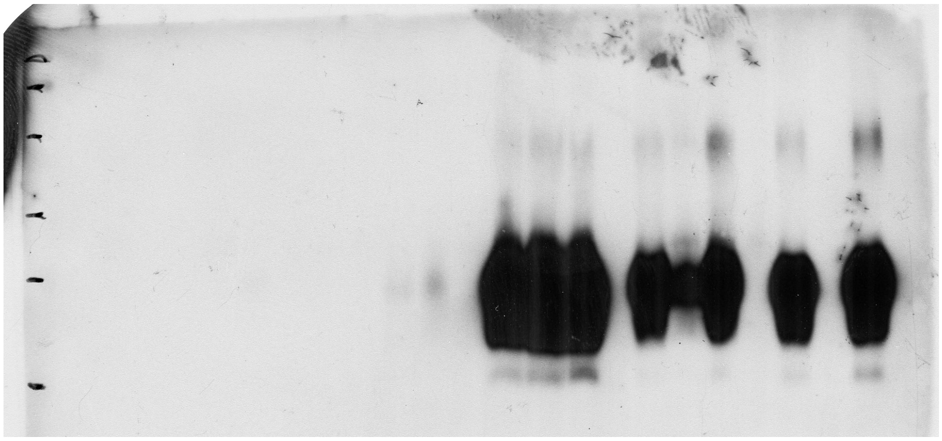

Supplementary Fig. 2 Uncropped Western blot. This is the uncropped Western blot of Supplementary Figure 1a.

Supplementary Figure 3

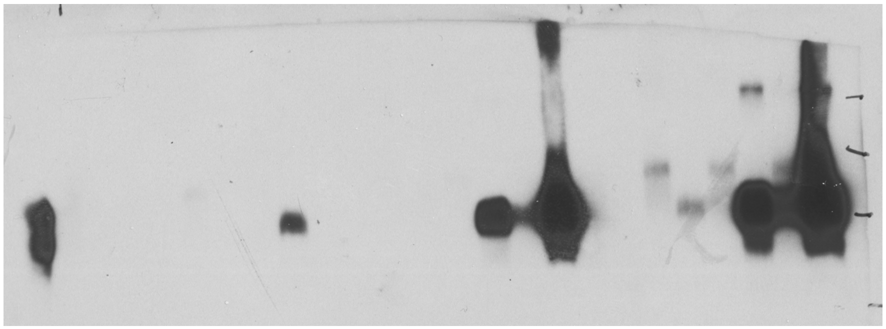

Supplementary Fig. 3 Uncropped Western blot. This is the uncropped Western blot of Figure 1a.

Supplementary Figure 4

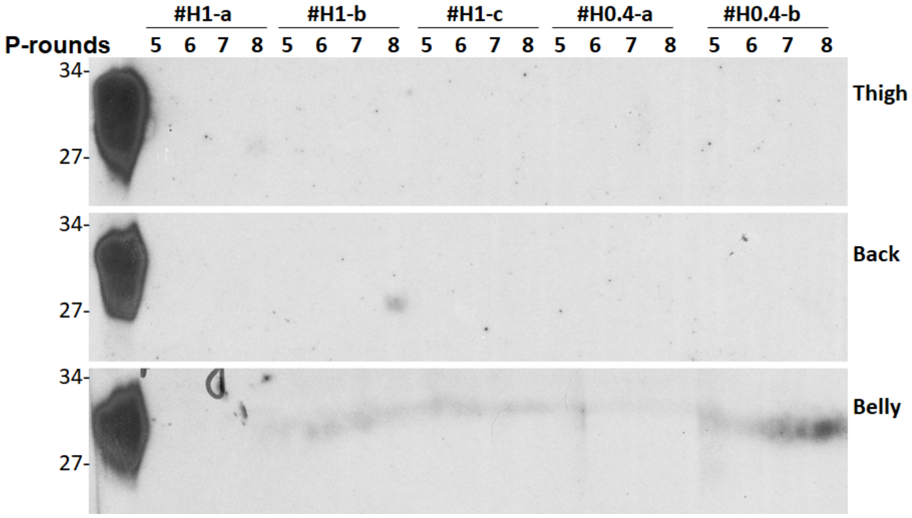

Supplementary Fig. 4 Representative Western blot analysis of skin PrP^{Sc} amplified by sPMCA. Amplified PrP^{Sc} from the thigh, back, and abdominal skin of hamsters sacrificed at 0.4 (n=2) and 1 (n=3) wpi of 263K prions using eight rounds of sPMCA. P-rounds: number of sPMCA rounds. All samples were treated with PK at 100 μg/ml except for the samples in the first lane of each

blot that were used as a non-PK treatment control. The blots were probed with 3F4. The molecular weight markers are shown in kDa on the left side of the blots.

Supplementary Figure 5

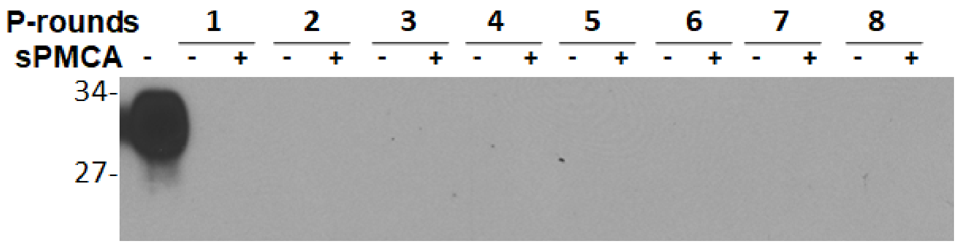

Supplementary Fig. 5 Representative Western blotting of back skin samples from the negative controls. The back skin samples from the negative control hamsters inoculated with PBS and housed separately from 263K-inoculated hamsters were subjected to eight rounds of sPMCA and then to Western blotting. All samples were treated with PK at 100 μg/ml except for the sample in lane 1 that was used as a negative control. P-rounds: number of sPMCA rounds. The blot was probed with the 3F4 antibody and it is a reprehensive of three different skin areas from three animals. The molecular weight markers are shown in kDa on the left side of the blot.

Supplementary Figure 6

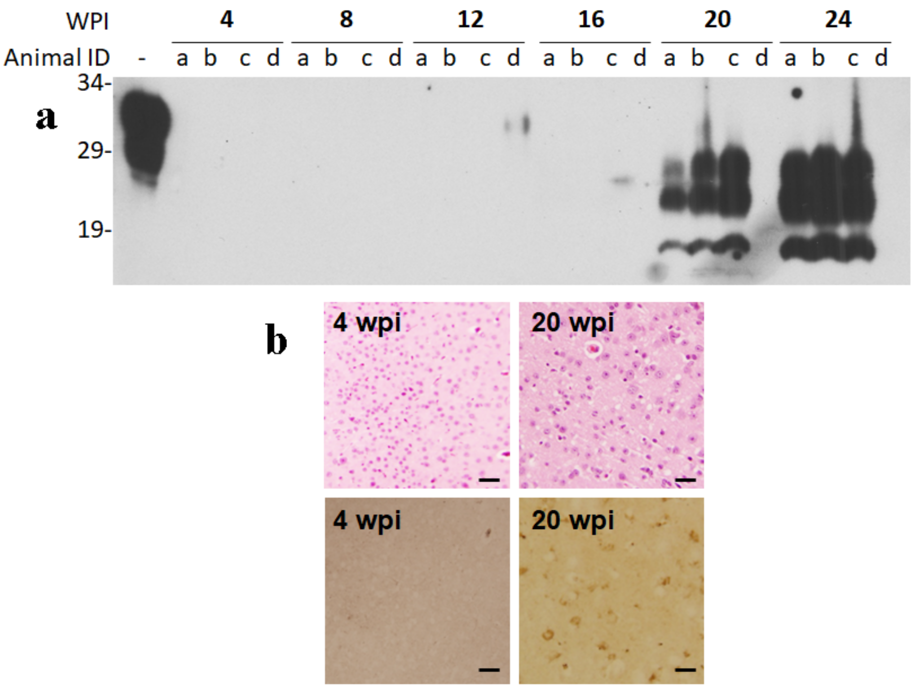

Supplementary Fig. 6 Western blotting, immunohistochemistry and Hematoxylin & Eosin staining of the brain of infected Tg40h mice. (***a***) Representative Western blotting of PrP^{Sc} in the brain of humanized Tg40h mice inoculated with sCJDMM1 brain homogenate euthanized at 4, 8, 20, 22, or 24 weeks post-inoculation (wpi). Animal ID: a, b, and c represent Tg mice that were inoculated with sCJDMM1 brain homogenate while d represents Tg mice that were inoculate with PBS at each time point. The blot was probed with 3F4 and it is representative of three independent experiments. Molecular weight markers are shown in kDa on the left side of each blot. (***b***) Hematoxylin & Eosin (H&E) staining and immunohistochemistry of brain sections with the 3F4 antibody from humanized Tg40h mice inoculated with sCJDMM1 brain homogenate euthanized at 4 or 20 wpi. Bar = 50 µm.

Supplementary Figure 7

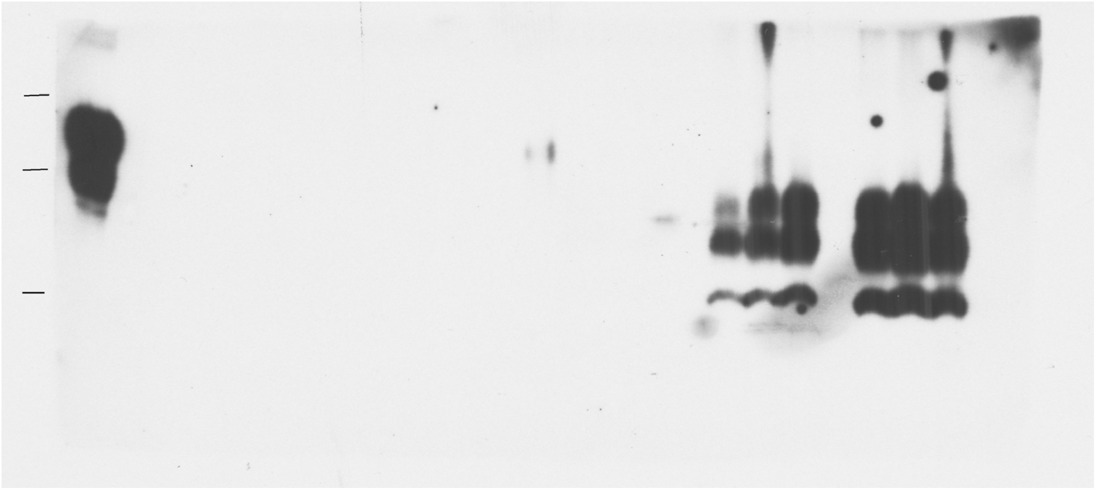

Supplementary Fig. 7 Uncropped Western blot. This is the uncropped Western blot of Supplementary Figure 6a.

Supplementary Figure 8

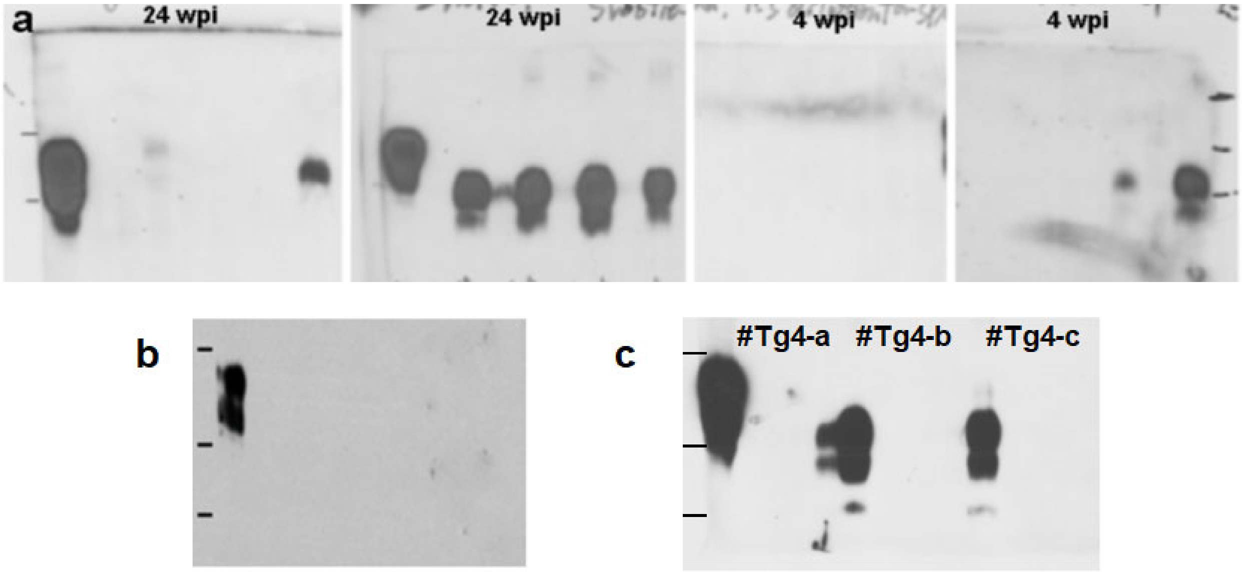

Supplementary Fig. 8 Uncropped Western blots. These are the uncropped Western blots of Figure 4.

Supplementary Figure 9

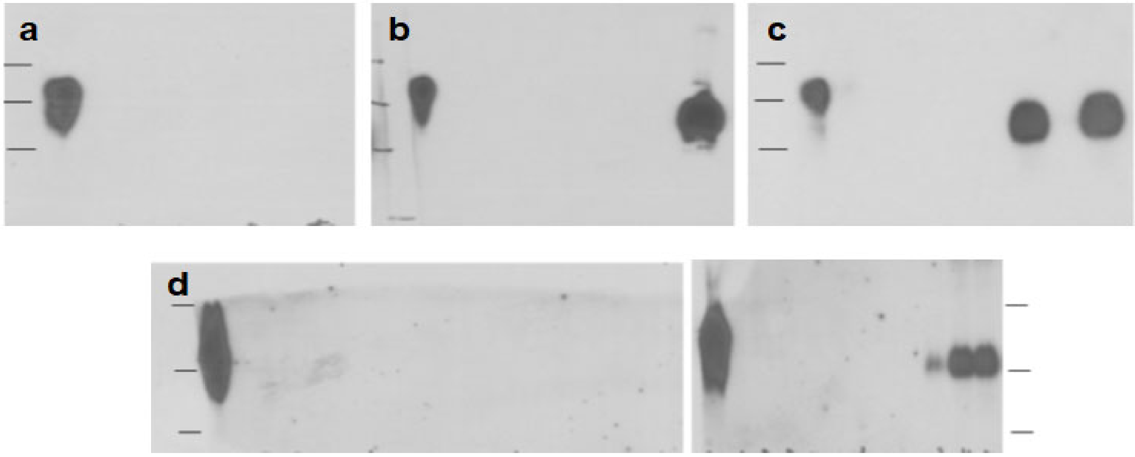

Supplementary Fig. 9 Uncropped Western blots. These are the uncropped Western blots of Figures 7a-d.
