# Supplementary material for: Early preclinical detection of prions in the skin of prion-infected animals
Source: Nat Commun. 2019 Jan 16;10:247. doi: 10.1038/s41467-018-08130-9 (PMC6335425; doi:10.1038/s41467-018-08130-9)
Supplement: Supplementary file 3 — Reporting Summary [file 41467_2018_8130_MOESM3_ESM.pdf]

nature research

Corresponding author(s): Wen-Quan Zou, Qingzhong Kong, Li Cui, Byron Caughey

Reporting Summary

Nature Research wishes to improve the reproducibility of the work that we publish. This form provides structure for consistency and transparency in reporting. For further information on Nature Research policies, see Authors & Referees and the Editorial Policy Checklist.

Statistical parameters

When statistical analyses are reported, confirm that the following items are present in the relevant location (e.g. figure legend, table legend, main text, or Methods section).

n/a	Confirmed	
☐	☒	The exact sample size (*n*) for each experimental group/condition, given as a discrete number and unit of measurement
☐	☒	An indication of whether measurements were taken from distinct samples or whether the same sample was measured repeatedly
☐	☒	The statistical test(s) used AND whether they are one- or two-sided<br>*Only common tests should be described solely by name; describe more complex techniques in the Methods section.*
☐	☒	A description of all covariates tested
☐	☒	A description of any assumptions or corrections, such as tests of normality and adjustment for multiple comparisons
☐	☒	A full description of the statistics including central tendency (e.g. means) or other basic estimates (e.g. regression coefficient) AND variation (e.g. standard deviation) or associated estimates of uncertainty (e.g. confidence intervals)
☐	☒	For null hypothesis testing, the test statistic (e.g. *F*, *t*, *r*) with confidence intervals, effect sizes, degrees of freedom and *P* value noted<br>*Give P values as exact values whenever suitable.*
☒	☐	For Bayesian analysis, information on the choice of priors and Markov chain Monte Carlo settings
☒	☐	For hierarchical and complex designs, identification of the appropriate level for tests and full reporting of outcomes
☒	☐	Estimates of effect sizes (e.g. Cohen's *d*, Pearson's *r*), indicating how they were calculated
☐	☒	Clearly defined error bars<br>*State explicitly what error bars represent (e.g. SD, SE, CI)*

Our web collection on statistics for biologists may be useful.

Software and code

Policy information about availability of computer code

Data collection	Software used for RT-QuIC data collection is BMG LABTECH Omega.
Data analysis	We used the Excel software for data analysis and making graphs and Epson expression 1680 for scanning protein bands on Western blots and UN-SCAN-IT for densitometric analysis.

For manuscripts utilizing custom algorithms or software that are central to the research but not yet described in published literature, software must be made available to editors/reviewers upon request. We strongly encourage code deposition in a community repository (e.g. GitHub). See the Nature Research guidelines for submitting code & software for further information.

Data

Policy information about availability of data

All manuscripts must include a data availability statement. This statement should provide the following information, where applicable:

- Accession codes, unique identifiers, or web links for publicly available datasets
- A list of figures that have associated raw data
- A description of any restrictions on data availability

The data that support the finding of this study are available from WQZ upon reasonable request. A reporting summary for this Article is available as a Supplementary Information file.

Field-specific reporting

Please select the best fit for your research. If you are not sure, read the appropriate sections before making your selection.

☒ Life sciences ☐ Behavioural & social sciences ☐ Ecological, evolutionary & environmental sciences

For a reference copy of the document with all sections, see nature.com/authors/policies/ReportingSummary-flat.pdf

Life sciences study design

All studies must disclose on these points even when the disclosure is negative.

Sample size	We think that the sample sizes are sufficient because for each time points we have 3-4 animals and for each animal, we have three different skin samples. Moreover, we have two types of animal models. Most importantly, the hamster experiment results were also confirmed by the another set of experiment which was done in the second independent laboratory.
Data exclusions	No data were excluded.
Replication	All our data were repeated at least for three times. As mentioned above, our hamster experiments were confirmed at the second independent laboratory.
Randomization	Our study was performed with two types of animal models and were well-desired with negative controls in each steps. So, all covariates were well controlled.
Blinding	The investigators were blinded without knowing which ones were positive and which ones were negative. All samples were coded.

Reporting for specific materials, systems and methods

Materials & experimental systems

n/a	Involved in the study	
☒	☐	Unique biological materials
☐	☒	Antibodies
☒	☐	Eukaryotic cell lines
☒	☐	Palaeontology
☐	☒	Animals and other organisms
☒	☐	Human research participants

Methods

n/a	Involved in the study	
☒	☐	ChIP-seq
☒	☐	Flow cytometry
☒	☐	MRI-based neuroimaging

Antibodies

Antibodies used	Two commercial antibodies were used in this study: the monoclonal antibody against prion protein 3F4: MAB1562, Chemicon International In, Burlington, MD, USA; Sheep anti-mouse IgG conjugated with horseradish peroxidase as the secondary antibody (ACiiiP, Chemicon International, Inc, Burlington, MD, USA).
Validation	The two antibodies have been well-validated by many laboratories and shown in the website of the company and the literature. Here are the links: http://www.emdmillipore.com/US/en/product/Anti-Prion-Protein-Antibody-a.a.-109-112-clone-3F4,MM_NF-MAB1562; http://www.emdmillipore.com/US/en/product/Sheep-Anti-Mouse-IgG-Antibody-Species-absorbed-Antibody-HRP-conjugate,MM_NF-AC111P

Animals and other organisms

Policy information about studies involving animals; ARRIVE guidelines recommended for reporting animal research

Laboratory animals	Syrian golden hamster were used at their age 2 weeks with sexes for inoculation of scrapie. Also humanized trangenic FVB mice expressing human prion protein with both sexes were used at 4 weeks of age for inoculation of human prions.
Wild animals	No wild animals were used in this study.
Field-collected samples	No field-collected samples were used in this study.
